# The Pattern of Thyroid Malignancy and Its Associated Characteristics Among United Arab Emirates Population With More Focus on Patients in the Bethesda III Category

**DOI:** 10.7759/cureus.23321

**Published:** 2022-03-19

**Authors:** Mahmoud A Kiblawi, Kashif Hafeez, Shahed K Lami, Omar A Al Teneiji, Abdullah N Al Mubarak, Thaer K Swaid, Sundos A Ahmed, Roaa S Alabiri, Ruba S Alabiri

**Affiliations:** 1 Department of Internal Medicine, Sheikh Shakhbout Medical City, Abu Dhabi, ARE; 2 Department of Endocrinology, Shiekh Shakbout Medical City, Abu Dhabi, ARE; 3 Department of Emergency Medicine, Rashid Hospital, Dubai, ARE; 4 Department of Neurology, Sheikh Shakhbout Medical City, Abu Dhabi, ARE; 5 Department of Internal Medicine, Gul Medical University, Ajman, ARE; 6 Department of Internal Medicine, Prince Sultan Military Medical City, Riyadh, SAU

**Keywords:** ultrasound score, thyroid nodule, goitre, bethesda, thyroid malignancy

## Abstract

Introduction: Thyroid cancer is the most prevalent endocrine cancer worldwide. It is the second most common type of cancer among United Arab Emirates (UAE) women and ranks as the sixth most common type of cancer overall among the UAE population. There are limited studies in the UAE related to thyroid malignancy. This study aimed to determine the pattern of thyroid malignancy among the UAE population and its associated characteristics, with more emphasis on patients categorized as Bethesda III by cytopathology, and furthermore, to determine the significance of advanced diagnostic methods in the assessment of thyroid nodules.

Methods: A retrospective review of the electronic medical charts of adult patients (age 18 and above) who were diagnosed with a thyroid nodule by ultrasound during the years 2019 and 2020. It is a comparative study of different variables associated with thyroid nodules and thyroid malignancy.

Results: A total of 1072 patients were diagnosed with thyroid nodules upon initial ultrasound. We had 174 patients diagnosed with thyroid malignancy, constituting 16% (95% CI 0.14-0.19) of the total study population. 78% of the thyroid malignancy patients were women as compared to men, and this difference was statistically significant (p=0.042). Non-UAE nationals comprised 61% of the population diagnosed with thyroid malignancy (95% CI 1.37-2.68). Malignancy was found to be more common in patients with multinodular goiter, in the 30 to 39-year age group, and in patients with high ultrasound and Bethesda grades. From the total study population, 140 patients had cytology reports in the Bethesda III category. Thyroid malignancy was found in 30 patients with Bethesda III, and this comprised 17% of the total population who were diagnosed with thyroid malignancy.

Conclusion: Despite being a single-center study, it highlights the percentage of thyroid malignancy and its associated factors among the UAE population. Thyroid ultrasound grading and Bethesda classification guide physicians in risk stratification, but it remains challenging in patients who fall into the Bethesda III category. Intervention versus regular follow-up should not depend on a single value but on the overall clinical picture and the use of advanced diagnostic methods.

## Introduction

Thyroid cancer is the most prevalent endocrine cancer and is the fifth most common malignancy in women worldwide [1-3]. It was reported in 2015 that 3.2 million people worldwide have thyroid cancer [4]. The overall crude prevalence of thyroid cancer among patients in the United Arab Emirates (UAE) over a four-year period (2011-2015) was 10.1% [5]. It is the second most common type of cancer among UAE women and ranks as the sixth most common type of cancer overall among the UAE population [5,6].

Many risk factors are associated with thyroid cancer, including age, female gender, endemic goitre, genetic susceptibility, familial thyroid cancer, obesity, and radiation exposure [7,8]. Thyroid cancer most commonly occurs between 45 and 54 years of age and its incidence varies by ethnicity/race [1,3]. Women are affected three times more often than men, but the death rate is similar in both genders at approximately 0.5 cases per 100,000 people [3,9].

The clinical importance of thyroid nodules rests with the need to exclude thyroid cancer, which occurs in 7-15% of cases depending on gender, age, and other associated risk factors [10]. There are two ultrasound scoring systems used for thyroid nodule classification: (1) Thyroid Imaging Reporting and Data System (TI-RADS) proposed by the American College of Radiology (ACR), and (2) "U" classification developed by the British Thyroid Association (BTA) [8,11]. These methods stratify the estimated risk of cancer in thyroid nodules and select those nodules needing to undergo fine-needle aspiration (FNA) [8,11]. Reporting of FNA specimens is done using the Bethesda System for Reporting Thyroid Cytopathology (TBSRTC), which is divided into six categories according to the risk of malignancy associated with each diagnostic category [12].

Thyroid malignancy is common among the UAE population, but we have limited studies and data reported. The aim of this research is to: (1) estimate the percentage of thyroid malignancy among the UAE population diagnosed with thyroid nodules; (2) study the demographics of the patient population; (3) identify the associated characteristics of benign and malignant thyroid nodules; and (4) determine the significance of the advanced diagnostic methods and scoring systems in assessing thyroid nodules. Lastly, we want to raise awareness about the pattern of thyroid malignancy among patients whose cytology is categorized as Bethesda III.

"This article was previously presented as an oral presentation at the nineth International Oncology Conference, Abu Dhabi, UAE, on December 17, 2021."

## Materials and methods

Design, setting, and data review

This is a retrospective review of the electronic medical records of adult patients (age 18 and above) who had ultrasounds of their thyroid, parathyroid, and neck during the years 2019 and 2020 (n=1636). Patients who had normal studies and below 18 years of age (n = 564) were excluded from the study. The study initially started at Mafraq Hospital, Abu Dhabi, UAE, but has now been transferred to Sheikh Shakhbout Medical City (SSMC).

Several factors were studied among patients diagnosed with thyroid nodules by ultrasound. In patients diagnosed with multiple thyroid nodules, we selected the nodule with the highest "TIRADS" or "U" score. Others who had unscored thyroid nodules were labelled as unclassified. As this is a retrospective study, FNA was indicated based on the referring physician, the endocrinologist, or the general surgeon. The final cytopathology results were categorized according to the Bethesda system. We focused more on the pattern of thyroid malignancy among the UAE population within the Bethesda III category. The demographics and characteristics of patients diagnosed with thyroid malignancy were identified and analyzed. 

Ethical approval was obtained from the SSMC research ethical committee to access patient medical charts and retrieve the data. As this is a retrospective study with no direct patient participation, verbal and written informed consent were not obtained from the patients.

Statistical analysis

This is a descriptive analysis of the data collected in patients with thyroid nodules at initial examination. The percentage of thyroid malignancy in the total study population was reported with a 95% confidence interval (CI). Using the SPSS software, the Chi-Square test was used to determine the statistical significance of the difference in thyroid cancer among the gender and nationality groups. We classified patients into five different age groups and determined in which age-adjusted group thyroid malignancy was most commonly found. Moreover, we focused on assessing the pattern of thyroid malignancy based on the demographics, patient characteristics, ultrasound scoring system, and Bethesda classification, especially those classified as category III.

## Results

Total population

A total of 1072 patients aged 18 years old and above were found to have thyroid nodules during an ultrasound done for thyroid, parathyroid, and neck during the years 2019 to 2020 (Table 1). The majority of the patients were aged between 30 and 49 years, and 83% of the total study population were women (n=890; Table 1).

**Table 1:** Demographics of the total study population

Total study population demographics	Total patients (n=1072)	Thyroid malignancy (n=174)
Gender	Male n=182 (17%)	Male n=39 (22%)
	Female n=890 (83%)	Female n=135 (78%)
Nationality	UAE national n=561 (52%)	UAE national n=67 (39%)
	Non-national n=511 (58%)	Non-national n=107 (61%)
Age (years)	(<30) n=131 (12%)	(<30) n=30 (17%)
	(30-39) n=295 (28%)	(30-39) n=60 (34.5%)
	(40-49) n=301 (28%)	(40-49) n=46 (26%)
	(50-59) n=173 (16%)	(50-59) n=25 (14%)
	(≥60) n=172 (16%)	(≥60) n=13 (7.5%)

**Figure 1:**
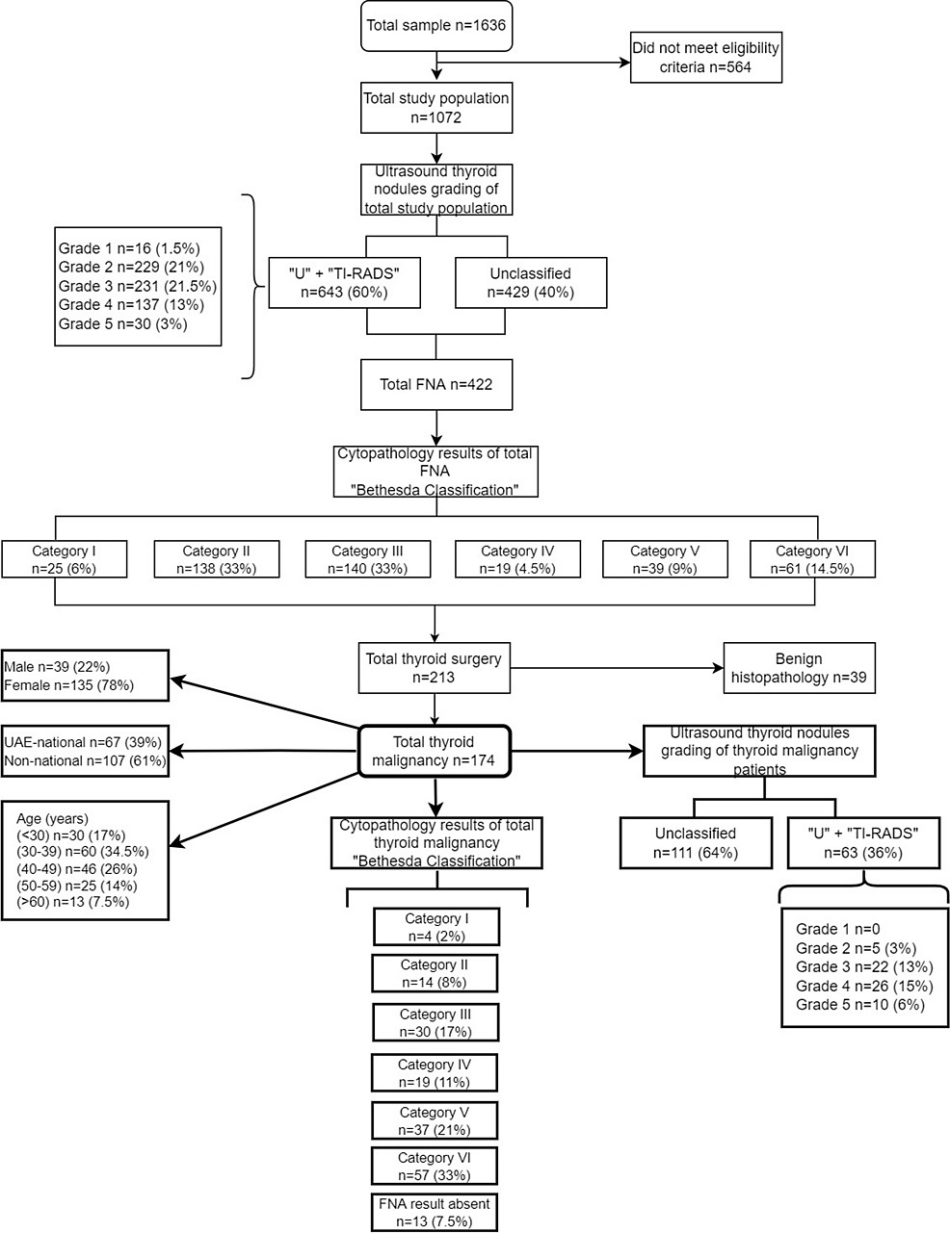
The percentage of thyroid malignancy among UAE population

FNA was performed in 39% of the total study population (n=422). Among those, 213 patients underwent thyroid surgery (Figure 1). We had 174 patients diagnosed with thyroid malignancy, constituting 16% of the total study population (95% CI 0.14-0.19; Figure 1 and Table 2). Malignancy was found in 78% of women (135) as compared to men, and this difference was statistically significant (p=0.042). Thyroid malignancy was most common among patients in the 30 to 39-year age group (Figure 1). There was not much difference in nationality in the total study population, but among patients diagnosed with thyroid malignancy, 61% were non-UAE nationals (n=107; 95% CI 1.37-2.68; Figure 1). The difference observed among the ethnic backgrounds was statistically significant (p=0.00), even when gender is taken as a cofounding variable.

**Table 2:** Bethesda classification of the total study population

Bethesda classification			
Category	Total FNA (n=422)	Malignancy (n=174)	Non-malignancy post-surgery (n=39)
I	n=25 (6%)	n=4 (2%)	n=1 (2.5%)
II	n=138 (33%)	n=14 (8%)	n=12 (31%)
III	n=140 (33%)	n=30 (17%)	n=16 (41%)
IV	n=19 (4.5%)	n=19 (11%)	n=0
V	n=39 (9%)	n=37 (21%)	n=1 (2.5%)
VI	n=61 (14.5%)	n=57 (33%)	n=1 (2.5%)
FNA result absent		n=13 (7.5%)	n=8 (20.5%)

**Figure 2:**
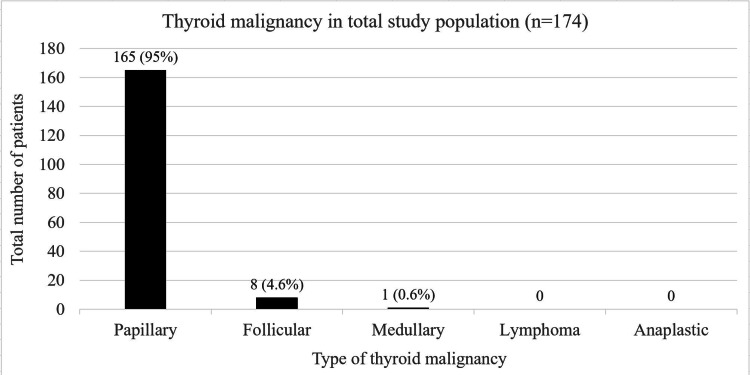
Types of thyroid malignancy from the total study population

**Figure 3:**
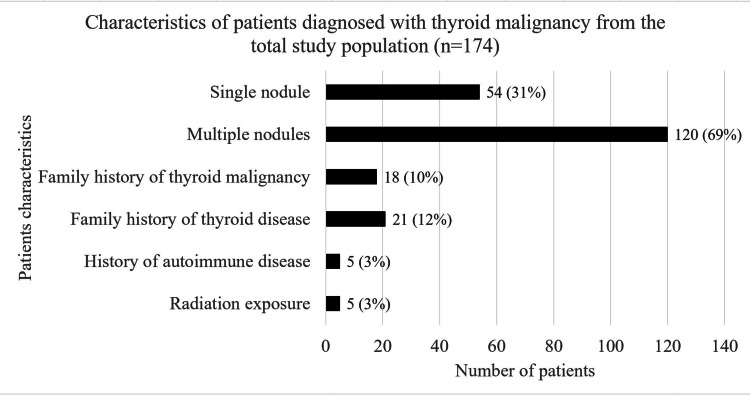
Thyroid malignancy patients characteristics - total study population

From the total study population, 95% of the patients (n=165) were diagnosed with papillary type thyroid malignancy (Figure 2). We also studied several characteristics of thyroid malignancy patients (Figure 3). Upon initial ultrasound evaluation, it was found that 31% had a single nodule (n=54) and the remaining 69% were diagnosed with multinodular goitre (n=120). Furthermore, 13% of our patients with thyroid malignancy had a co-existing malignancy (n=22).

Bethesda III

A total of 140 patients from the total study population were classified in the Bethesda III Category. The ratio of female to male gender was 4.6 to 1 (Table 3). Forty-six patients diagnosed with Bethesda III underwent thyroid surgery (Table 3). Among those, 30 patients were diagnosed with thyroid malignancy, and this comprises 17% of the total thyroid malignancy patients. Again, it was found to be more common among women and in the 30-39 age group of patients (Table 3).

**Table 3:** Demographics of Bethesda III patients

Bethesda III demographics	Total patients (n=140)	Thyroid malignancy (n=30)
Gender	Male n=25 (18%)	Male n=5 (17%)
	Female n=115 (82%)	Female n=25 (83%)
Nationality	UAE national n=72 (51%)	UAE national n=15 (50%)
	Non-national n=68 (49%)	Non-national n=15 (50%)
Age (years)	(<30) n=12 (8.5%)	(<30) n=5 (17%)
	(30–39) n=34 (24%)	30–39) n=9 (30%)
	(40–49) n=47 (33.5%)	(40–49) n=7 (23%)
	(50–59) n=18 (13%)	(50–59) n=5 (17%)
	(≥60) n=29 (21%)	(≥60) n=4 (13%)
Total patients who underwent thyroid surgery	Surgery (n=46)	
Type of thyroid malignancy		Papillary n=26 (87%)
		Follicular n=4 (13%)

**Figure 4:**
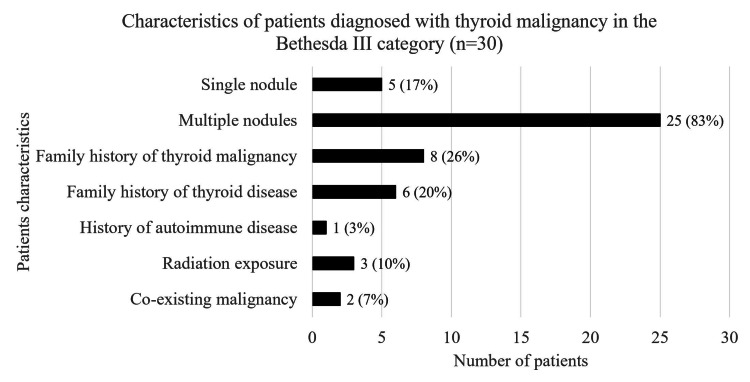
Thyroid malignancy patients characteristics - Bethesda III

High malignancy rates in the Bethesda III patients had an initial ultrasound grade of 3 and 4 as classified by BTA and TI-RADS (Table 4). We observed that 83% of the Bethesda III patients had multinodular goitre (n=25) upon initial ultrasound evaluation (Figure 4). Among the 30 patients, we had a single patient with co-existing breast cancer and another female patient with endometrial cancer.

**Table 4:** Ultrasound grading in the Bethesda III population

Bethesda III ultrasound scoring system – “U” and “TI-RADS”			
Classification	Grading	Total Bethesda III (n=140)	Malignancy (n=30)
“U” + “TI-RADS”	Grade 1	n=0	n=0
	Grade 2	n=8 (6%)	n=1 (3%)
	Grade 3	n=55 (39%)	n=10 (33%)
	Grade 4	n=39 (28%)	n=10 (33%)
	Grade 5	n=11 (8%)	n=1 (3%)
Unclassified		n=27 (19%)	n=8 (27%)

## Discussion

The incidence of thyroid cancer has increased in the past few decades [1,13-15]. This is believed to be due to more patients' access to healthcare, the implementation of new diagnostic approaches, and the increased use of ultrasound for better identification of thyroid nodules [13,14]. Our study showed that the percentage of thyroid malignancy in those who underwent thyroid surgery based on the clinical presentation, ultrasound findings, and cytopathology was 16%. This is believed to be high among the population of the UAE, similar to other studies conducted in the UAE and other parts of the Middle East [6,16]. Despite a higher incidence of malignancy being found among non-UAE nationals, the rate of malignancy among UAE nationals was 39%. This is considered high as the ratio of Emirati to non-nationals is 1:4 [17]. This can be explained by the fact that nationals had better health access than other residents from other nationalities.

Most of our thyroid malignancy patients were female, which means they were affected three times more than the male gender. This further confirms that women carry a higher risk of developing thyroid cancer [3,9]. However, this was not the case in other studies in the UAE, where a higher incidence of malignancy was found in the male gender of Asian descent [6]. Interestingly, thyroid malignancy remains higher among younger age groups, and our study showed that most patients were below the age of 49. Similar findings were observed in other studies conducted in the UAE [6]. As mentioned earlier, the use of advanced diagnostic methodology and the availability of access to the medical system can be the reason for the early identification of thyroid cancer. Moreover, this can be a scope to study further to determine if younger age groups have specific risk factors leading to thyroid malignancy.

According to the British Thyroid Guidelines, patients with ultrasound scoring grades of three or more should be referred for FNA [8]. Furthermore, patients' associated risk factors and nodule characteristic features can also guide physicians to determine if FNA is warranted [18]. In our study, high malignancy rates scored a grade of three or above, as classified by BTA and TI-RADS. However, we still have high rates of unclassified ultrasound scores. This comprised 40% of the total population (n=429), and malignancy was found in 26% of these patients (n=111). This proves the importance of implementing this scoring system in radiology departments during the evaluation of thyroid nodules.

TBSRTC is a reliable system used in categorizing thyroid nodules cytology with risk of malignancy [19]. It is divided into six categories: (i) nondiagnostic or unsatisfactory; (ii) benign (malignancy risk 0-3%); (iii) atypia of undetermined significance (AUS) or follicular lesion of undetermined significance (FLUS) (malignancy risk 5-15%); (iv) follicular neoplasm or suspicious for a follicular neoplasm (malignancy risk 15-30%); (v) suspicious for malignancy (malignancy risk 60-75%); and (vi) malignant (malignancy risk 97-99%) [12]. Surgical intervention is required in patients diagnosed with Bethesda IV-VI [12]. Of the 422 patients who underwent FNA, 66% (n=278) were categorized as Bethesda II and III. This is similar to findings observed in other studies [5,19]. More than 93% of the patients who had Bethesda IV and above were diagnosed with thyroid malignancy. Patients with benign histopathology (n=39) mainly had Bethesda below IV, which is reassuring. The decision for surgery, especially in patients with intermediate scores, was based on the ultrasound nodule characteristics, patient preference, and multidisciplinary team decision. We assume that some patients in the low-risk groups may have opted for thyroid surgery for different causes, such as cosmetic or pressure symptoms. Patients with intermediate scores were advised for observation, regular follow-up, and repeat FNA as per the BTA guidelines [20].

The risk of thyroid malignancy in patients categorized as Bethesda III is 5-15% [19]. We had 174 patients with thyroid nodules diagnosed with thyroid malignancy, and among those, 30 patients had Bethesda III, which accounts for 17% of the patients. It is a significant number, although not all patients had thyroid surgery. It was more common in the female gender, the age group of 30-39, and patients with initial ultrasound findings of multinodular goitre. Most of the patient’s histopathology was reported as papillary thyroid carcinoma, consistent with other regions' data [6].

A meta-analysis and a systemic review study have reported that single nodules carry a higher risk of thyroid malignancy than multinodular goitre [6,21]. Still, in our study, 69% of the patients with thyroid malignancy had multiple nodules on initial presentation. This can confirm the hypothesis that iodine-deficient areas such as the UAE cause multinodular goitre, further posing a risk of malignancy among our population. This is contrary to the United States of America (USA), where thyroid malignancy is higher in patients with single nodules [21]. It is reported in the literature that the presence of a co-existing malignancy can carry a risk factor for developing thyroid malignancy. Several factors have been identified which play a role in thyroid and breast cancer concurrence [22,23]. In our study, 13% of the thyroid malignancy patients had co-existing malignancies, but these were not further studied nor analyzed to determine if any clear causation exists.

Strengths and limitations

This is a large sample size of comprehensive patient data to determine the percentage and features of thyroid malignancy among the UAE population. This is considered the first study in the UAE to target patients in the Bethesda III category. Not many studies have been conducted in our region, and our significant findings can open the scope for further studies relevant to thyroid cancer. This includes associated risk factors, especially in younger age groups, the role of multinodular goitre, and the management approach toward patients categorized as Bethesda III. The study does have several limitations that could influence the results. In this single-center study, detailed nodule characteristics were not identified, and many thyroid imaging scores were unclassified. Also, patients may have lost to follow-up or lacked access to a tertiary hospital. Others may have ended up in other centers for a second opinion or surgery. These factors play a role in the outcome of the study.

## Conclusions

Our study showed that thyroid malignancy occurs in a high proportion among the UAE population. It was observed more in patients with multinodular goitre, younger age group, female gender, and non-UAE national. A significant number of patients were diagnosed with thyroid malignancy in the Bethesda III category, despite the fact that not all patients underwent surgical intervention. If surgery is delayed, we recommend a more frequent follow-up for this group of patients. This grey zone area remains a challenge in thyroid malignancy, and further studies are required in this group of patients.

Thyroid malignancy was found more frequently in patients with an ultrasound grade above three and in the Bethesda IV-VI categories. Therefore, it is highly recommended to implement advanced diagnostic methods and scoring systems in hospitals, providing adequate training for physicians and sonographers. The management of thyroid nodules is always based on the whole clinical picture rather than a single variable. It is essential to build a good rapport with patients and involve them in the decision-making.

## References

[1] (2021). Cancer stat facts: thyroid cancer. https://seer.cancer.gov/statfacts/html/thyro.html.

[2] Brown RL, de Souza JA, Cohen EE (2011). Thyroid cancer: burden of illness and management of disease. J Cancer.

[3] Pellegriti G, Frasca F, Regalbuto C, Squatrito S, Vigneri R (2013). Worldwide increasing incidence of thyroid cancer: update on epidemiology and risk factors. J Cancer Epidemiol.

[4] (2016). Global, regional, and national life expectancy, all-cause mortality, and cause-specific mortality for 249 causes of death, 1980-2015: a systematic analysis for the Global Burden of Disease Study 2015. Lancet.

[5] Alseddeeqi E, Baharoon R, Mohamed R, Ghaith J, Al-Helali A, Ahmed LA (2018). Thyroid malignancy among patients with thyroid nodules in the United Arab Emirates: a five-year retrospective tertiary Centre analysis. Thyroid Res.

[6] Al-Zaher N, Al-Salam S, El Teraifi H (2008). Thyroid carcinoma in the United Arab Emirates: perspectives and experience of a tertiary care hospital. Hematol Oncol Stem Cell Ther.

[7] (2021). Thyroid cancer: risk factors. https://www.cancer.net/cancer-types/thyroid-cancer/risk-factors.

[8] Perros P, Boelaert K, Colley S (2014). Guidelines for the management of thyroid cancer. Clin Endocrinol (Oxf).

[9] Popoveniuc G, Jonklaas J (2012). Thyroid nodules. Med Clin North Am.

[10] Haugen BR, Alexander EK, Bible KC (2016). 2015 American Thyroid Association Management Guidelines for adult patients with thyroid nodules and differentiated thyroid cancer: the American Thyroid Association Guidelines task force on thyroid nodules and differentiated thyroid cancer. Thyroid.

[11] Horvath E, Majlis S, Rossi R, Franco C, Niedmann JP, Castro A, Dominguez M (2009). An ultrasonogram reporting system for thyroid nodules stratifying cancer risk for clinical management. J Clin Endocrinol Metab.

[12] Cibas ES, Ali SZ (2017). The 2017 Bethesda system for reporting thyroid cytopathology. Thyroid.

[13] Morris LG, Sikora AG, Tosteson TD, Davies L (2013). The increasing incidence of thyroid cancer: the influence of access to care. Thyroid.

[14] Wiltshire JJ, Drake TM, Uttley L, Balasubramanian SP (2016). Systematic review of trends in the incidence rates of thyroid cancer. Thyroid.

[15] Olson E, Wintheiser G, Wolfe KM, Droessler J, Silberstein PT (2019). Epidemiology of thyroid cancer: a review of the national cancer database, 2000-2013. Cureus.

[16] El-Gammal AS, E-Balshy MA, Zahran KM (2019). Relationship between thyroid nodule size and incidence of thyroid cancer. Menoufia Med J.

[17] (2021). UAE population 1950-2021. https://www.macrotrends.net/countries/ARE/uae/population.

[18] Tamhane S, Gharib H (2016). Thyroid nodule update on diagnosis and management. Clin Diabetes Endocrinol.

[19] Alshaikh S, Harb Z, Aljufairi E, Almahari SA (2018). Classification of thyroid fine-needle aspiration cytology into Bethesda categories: an institutional experience and review of the literature. Cytojournal.

[20] Ho AS, Sarti EE, Jain KS (2014). Malignancy rate in thyroid nodules classified as Bethesda category III (AUS/FLUS). Thyroid.

[21] Brito JP, Yarur AJ, Prokop LJ, McIver B, Murad MH, Montori VM (2013). Prevalence of thyroid cancer in multinodular goiter versus single nodule: a systematic review and meta-analysis. Thyroid.

[22] Nielsen SM, White MG, Hong S (2016). The breast-thyroid cancer link: a systematic review and meta-analysis. Cancer Epidemiol Biomarkers Prev.

[23] Liu Y, Su L, Xiao H (2017). Review of factors related to the thyroid cancer epidemic. Int J Endocrinol.

